# Progranulin autoantibodies in systemic sclerosis and autoimmune connective tissue disorders: A preliminary study

**DOI:** 10.1002/iid3.270

**Published:** 2019-09-10

**Authors:** Philipp Klemm, Gunter Assmann, Klaus‐Dieter Preuss, Natalie Fadle, Evi Regitz, Thierry Martin, Michael Pfreundschuh, Lorenz Thurner

**Affiliations:** ^1^ Saarland University Medical School José Carreras Center for Immuno‐ and Gene Therapy and Internal Medicine I Homburg/Saar Germany; ^2^ Department of Rheumatology and Immunology Justus Liebig University Gießen, Campus Kerckhoff Bad Nauheim Germany; ^3^ CNRS, UPR3572, IBMC, Hopitaux Universitaires de Strasbourg Université de Strasbourg Strasbourg France

**Keywords:** autoimmune connective tissue disorders, progranulin, proinflammatory autoantibody, systemic sclerosis, TNF

## Abstract

**Objective:**

The present study aimed to investigate progranulin autoantibodies in systemic sclerosis and autoimmune connective tissue disorders. Progranulin is a physiologic tumor necrosis factor (TNF) antagonist. Progranulin antibodies decrease progranulin levels.

**Methods:**

Serum samples of 123 patients with systemic sclerosis and various autoimmune connective tissue disorders (Sjoegren's syndrome [SjS], mixed connective tissue disorder, polymyositis [PM] and dermatomyositis [DM], antiphospholipid syndrome [APLS], and undifferentiated connective tissue disease [UCTD]) were tested for progranulin antibodies using enzyme‐linked immunosorbent assay.

**Results:**

Progranulin antibodies were found in 34 of 123 (27.6%) patients at least once during their disease. In detail, 2 of 8 (25%) patients with limited cutaneous systemic sclerosis, 10 of 31 (32.3%) patients with diffuse cutaneous systemic sclerosis, 9 of 22 (40.9%) patients with SjS, 1 of 3 (33.33%) patients with mixed connective tissue disease, 4 of 33 (12.1%) patients with DM or PM, 6 of 15 (40%) patients with APLS, and 2 of 11 (18.2%) patients with UCTD were positive for progranulin antibodies during the course of disease.

**Conclusions:**

Progranulin antibodies are frequently present in patients with systemic sclerosis and other autoimmune connective tissue disorders. Despite the lack of specificity for a given autoimmune disease, progranulin antibodies might not only indicate a potential subtype but also play a pathogenic role in patients with autoimmune connective disorders. Given the important role of TNF‐α in inflammatory processes in autoimmune connective tissue disorders, progranulin antibodies might support the proinflammatory environment by neutralizing the TNF blocker progranulin.

## 1 INTRODUCTION

Recently, we identified progranulin autoantibodies (PGRN‐Abs) by a protein‐array screening of sera from patients with several primary systemic vasculitides. This finding was confirmed by enzyme‐linked immunosorbent assay (ELISA) using recombinant C‐terminally FLAG‐tagged and in HEK293 cells expressed human progranulin as bait. In extended screenings, PGRN‐Abs were also frequently detected in rheumatoid arthritis and systemic lupus erythematosus (SLE), but only very rarely (<1%) in healthy controls or in patients with sepsis or melanoma.^1^ Additionally, PGRN‐Abs could be found in inflammatory bowel disease^2^ and psoriatic arthritis.^3^ We found that PGRN‐Abs constitute a pathogenic role: PGRN‐Abs decrease progranulin plasma levels measured using ELISA and Western immunoblot.^1^, ^3^ In tumor necrosis factor α (TNF‐α)‐induced cytotoxicity assays, the addition of sera from healthy controls and PGRN Ab‐negative patients reduced TNFα‐induced cytotoxicity of WEHI‐S and HT‐1080 cells to a significantly higher degree than sera of PGRN Ab‐positive patients.^2^, ^3^, ^4^ Additionally, Western blot showed that the administration of recombinant PGRN antagonized the TNFα‐induced downmodulation of FOXP3 expression to a similar extent as the administration of etanercept. The TNFα‐inhibiting effect of PGRN was blocked by the administration of anti‐PGRN‐Fab.^2^ The occurrence of PGRN‐Abs is associated to an active disease state.^1^ As an underlying cause for autoimmunity to progranulin, we found the inactivated (pThr320) protein phosphatase 1 inducing an immunogenic hyperphosphorylation of progranulin at Ser81 to be the underlying cause of PGRN‐Abs. Additionally, this hyperphosphorylation prevents direct binding to and inhibition of tumor necrosis factor receptor 1 (TNFR1), TNFR2, and DR3 by PGRN and alters the conversion of progranulin into mature granulin motifs.^4^ Therefore, not only PGRN‐Abs restricts progranulin function but the cause for autoimmunity to progranulin itself limits progranulin function.

PGRN is a secreted precursor glycoprotein of 88 kDa, which consists of one N‐terminal signal peptide and seven granulin motifs, each containing 12 cysteins forming six disulfide bridges.^5^ The full‐length PGRN protein can be split into the individual granulin motifs, which have biological effects that are different from those of PGRN.^5^, ^6^ PGRN is expressed in leukocytes, neurons, neuroglia, chondrocytes, and epithelial cells.^7^ Thus, PGRN has various distinct functions. It is suspected to promote progression of various carcinomas by stimulating proliferation, inhibiting apoptosis, and promoting invasion.^8^ Furthermore, it plays a vital part in the progress of neurodegeneration in frontotemporal dementia frontotemporal lobar dementia (FTLD),^9^ cartilage development and degeneration, inflammation, and host defense.^8^, ^10^, ^11^ Progranulin also plays a major role after tissue injury. In the response phase after tissue injury, fibroblasts, endothelial cells, and infiltrating inflammatory cells highly increase PGRN expression.^5^

Of these, the role of PGRN in inflammation is of substantial interest. Moreover, PGRN is nowadays considered to be a key player in autoimmune diseases.^12^ PGRN is a strong anti‐inflammatory mediator acting through the inhibition of TNFR1 and TNFR2.^13^ The peptide chain consisting of granulin fragments F, A, and C with their linker regions was identified as the relevant ligand for TNFR1 and TNFR2 with an inhibitory effect. Subsequently, Atsttrin (antagonist of TNF‐TNFR‐signaling via targeting to TNFR) was engineered as a protein derived from PGRN. Atsttrin is constituted of half‐units of granulins A, C, and F plus linkers P3, P4, and P5.^14^ Studies indicated the engineered protein Atsttrin has similar anti‐inflammatory effects as PGRN. Furthermore, Atsttrin displays benefits over PGRN such as longer half‐life, higher efficacy, lower molecular weight, and most importantly no oncogenic effects.^15^ In preliminary studies, Atsttrin proofed to have therapeutic effect in models of inflammatory arthritis,^14^, ^15^ inflammatory bowel disease,^16^ and showed protective effects in bone healing and osteoarthritis.^17^

In consideration of this possible proinflammatory role of PGRN‐Abs and given the frequent occurrence of PGRN‐Abs in the course of SLE, the present study was aimed to examine PGRN‐Abs in serum of patients with systemic sclerosis and other autoimmune connective tissue disorders.

## 2 MATERIAL AND METHODS

When needed, patient's written informed consent was obtained. Serum samples were obtained at Saarland University Hospital in Homburg/Saar during the period from 2009 to 2011. Additional serum samples were provided by Strasbourg University Hospital (France). From several patients, multiple serum samples were obtained at different time points. If multiple serums samples were tested per patient, the patient was considered positive for PRGN‐Abs if at least one serum sample was positive. All sera were stored at −20°C until use.

### 2.1 ELISA for PGRN‐Ab

PGRN‐Ab ELISA was performed as described before.^1^

### 2.2 Statistical analysis

All statistical analyses were performed in SPSS Version 19.0 for Windows (IBM SPSS). *χ* ^2^ tests were done when appropriate.

## 3 RESULTS

Sera from 89 patients treated at Saarland University, Homburg, and from 34 patients treated at Strasbourg University Hospital were included in this study. Detailed clinical characteristics can only be provided from patients treated at Saarland University Hospital. The median age of patients was 59.0 years. For further patient characteristics see Table 1.

**Table 1:** Associations of PGRN‐Abs with clinical parameters

	lcSSc	dcSSc	SjS	MCTD	DM/PM	APLS	UCTD
Patients	N = 8	N = 31	N = 22	N = 3	N = 33	N = 15	N = 11
Missing clinical data	N = 0	N = 5	N = 9	N = 0	N = 6	N = 14	N = 0
Sex							
Male	0	9	0	0	9	0	2
Female	8	17	13	3	18	1	9
Median of age, y	70.0	63.50	51.0	50.0	62.0	55.0	47.0
PRGN‐Abs							
Yes	2	10	9	1	4	6	2
No	6	21	13	2	29	9	9
Established Abs	Anti‐CentromerB	Anti‐Scl70	Anti‐Ro (SSA), Anti‐La (SSB)	Anti‐U1	Anti‐Jo‐1	Anti‐CL, Anti‐b‐2GP1	Anti‐dsDNA, Anti‐Sm, Anti‐Ro, Anti‐La, Anti‐Jo1
Yes	6	11	10	3	3	1	4
No	2	15	3	0	24	0	7
Immunosuppressive therapy							
Yes	5	14	6	2	22	1	5
No	3	12	7	1	5	0	6

Abbreviations: APLS, antiphospholipid syndrome; DM/PM, dermatomyositis/polymyositis; lcSSc, limited cutaneous systemic sclerosis; dcSSc, diffuse cutaneous systemic sclerosis; MCTD, mixed connective tissue disease; SjS, Sjoegren's syndrome; UCTD, undifferentiated connective tissue disease.

**Figure 1:**
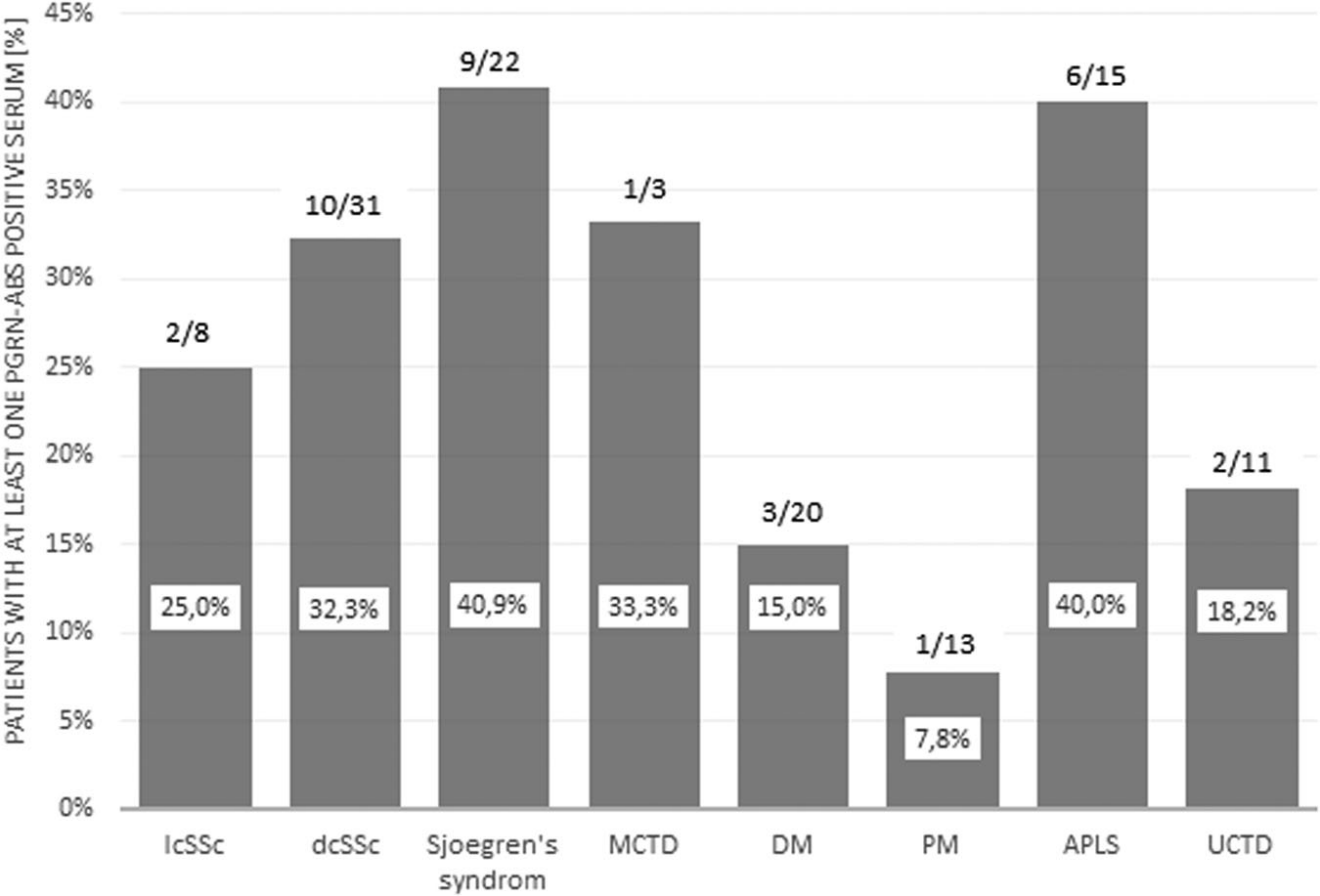
Occurrence of PGRN‐Abs in autoimmune connective tissue disorders. APLS, antiphospholipid syndrome; DM/PM, dermatomyositis/polymyositis; lcSSc, limited cutaneous systemic sclerosis; dcSSc, diffuse cutaneous systemic sclerosis; MCTD, mixed connective tissue disease; PGRN‐Abs, progranulin autoantibody; SjS, Sjoegren's syndrome; UCTD, undifferentiated connective tissue disease

Overall, progranulin antibodies were detected in 34 of 123 (27.6%) patients. Two of eight (25%) patients with limited cutaneous systemic sclerosis, 10 of 31 (32.3%) patients with diffuse cutaneous systemic sclerosis, 9 of 22 (40.9%) patients with Sjoegren's syndrome (SjS), 1 of 3 (33.33%) patients with mixed connective tissue disease, 4 of 33 (12.1%) patients with dermatomyositis or polymyositis, 6 of 15 (40.0%) patients with antiphospholipid syndrome, and 2 of 11 (18.2%) patients with undifferentiated connective tissue disease were positive for progranulin antibodies (Figure 1).

Moreover, a statistically significant association (*P* = .036) was detected between multiple serum samples obtained at Saarland University, Homburg at different points of time per individual patient and a positive PGRN‐Ab carrier state (Table 2).

**Table 2:** Association between single or multiple serum samples per individual patient and the occurrence of PGRN‐Abs

	PGRN‐Ab			
Single or multiple serum samples per individual patient	Negative	Positive	Total	*P* (*χ* ^2^)
Single sample	45	10	55	
Multiple samples	21	13	34	
Total	66	23	89	.036

## 4 DISCUSSION

This study revealed the frequent occurrence of PGRN‐Abs in the sera of patients with systemic sclerosis and other autoimmune connective tissue disorders, in addition to the previously described frequent occurrence of such antibodies in SLE. In consideration of the frequency of PGRN‐Abs in several systemic primary vasculitides,^1^ in rheumatoid arthritis,^1^ psoriatic arthritis,^3^ and inflammatory bowel disease,^2^ this clearly shows that the presence of PGRN‐Abs is not restricted to a particular autoimmune disease. In contrast, PGRN‐Abs have been absent or very infrequently detected in various control groups including healthy controls and patients with sepsis or melanoma. In the context of the reported PGRN‐neutralizing and thus putative proinflammatory effects of PGRN‐Abs, the present findings support the hypothesis that PRGN‐Ab represents a common proinflammatory stimulus in a wide spectrum of autoimmune diseases.

This finding could have clinical relevance because PGRN‐Ab serostatus could eventually be useful as a new biomarker for individualized therapeutic strategies. First, patients with PGRN‐Abs have as outlined above less anti‐TNF‐α capacity,^1^, ^2^, ^3^, ^4^ and could particularly profit from the administration of therapeutic TNF‐α blockers. Second, PGRN‐Ab serostatus might be useful as a predictive marker for the efficacy of B‐cell depleting therapies. In the present study, sequential serum samples obtained at different time points during the course of disease were available from a subgroup of patients. Interestingly, a statistically significant association was detected between multiple serum samples per individual patient and a positive PGRN‐Ab status during the course of disease (Table 2). This could be explained by seroconversions of PGRN‐Abs during the course of disease. Third, recently we identified pSer81‐PGRN as the carrier of autoimmunity against PGRN.^4^ PGRN could again be modified during the course of disease and thus, explain the seroconversion from positive to negative regarding PGRN‐Abs. Fourth, with the engineered PGRN‐analog on Atsttrin being therapeutically tested in various diseases^17^, ^18^ the presence and description of a preliminary incidence of PGRN‐Abs in various autoimmune diseases seem to be important. In this respect, further studies examining the frequency of PGRN‐Abs in larger cohorts of patients should be started. Additionally, a possible interaction between PGRN‐Abs and Atsttrin should be tested.

Taken together, given the important role of PGRN in various autoimmune diseases and the potential functional impact of PGRN‐Abs, our data support the idea of a substantial role of PGRN/PGRN‐Abs in systemic sclerosis and other autoimmune connective tissue disorders.

## CONFLICT OF INTERESTS

University of Saarland, Lorenz Thurner, Klaus‐Dieter Preuss, and Michael Pfreundschuh have applied for a relevant patent.

## DATA ACCESSIBILITY

The data that support the findings of this study are available from the corresponding author upon reasonable request as restrictions apply to the availability of these data, which were used under license from Saarland University, Homburg and Strasbourg University Hospital for this study.

## ETHICS STATEMENT

This study had been approved by the local ethical review board (“‘Ethikkommission der Ärztekammer des Saarlandes”’; Ethikantrag Nr. 242/11) and was conducted according to the Declaration of Helsinki. When needed, patient's written informed consent was obtained.
